# The Role of Dendritic Cells During Infections Caused by Highly Prevalent Viruses

**DOI:** 10.3389/fimmu.2020.01513

**Published:** 2020-07-16

**Authors:** Jorge A. Soto, Nicolas M. S. Gálvez, Catalina A. Andrade, Gaspar A. Pacheco, Karen Bohmwald, Roslye V. Berrios, Susan M. Bueno, Alexis M. Kalergis

**Affiliations:** ^1^Departamento de Genética Molecular y Microbiología, Facultad de Ciencias Biológicas, Instituto Milenio de Inmunología e Inmunoterapia, Pontificia Universidad Católica de Chile, Santiago, Chile; ^2^Departamento de Endocrinología, Facultad de Medicina, Instituto Milenio de Inmunología e Inmunoterapia, Pontificia Universidad Católica de Chile, Santiago, Chile

**Keywords:** dendritic cells, IFN, antiviral response, viruses, immune response

## Abstract

Dendritic cells (DCs) are a type of innate immune cells with major relevance in the establishment of an adaptive response, as they are responsible for the activation of lymphocytes. Since their discovery, several reports of their role during infectious diseases have been performed, highlighting their functions and their mechanisms of action. DCs can be categorized into different subsets, and each of these subsets expresses a wide arrange of receptors and molecules that aid them in the clearance of invading pathogens. Interferon (IFN) is a cytokine -a molecule of protein origin- strongly associated with antiviral immune responses. This cytokine is secreted by different cell types and is fundamental in the modulation of both innate and adaptive immune responses against viral infections. Particularly, DCs are one of the most important immune cells that produce IFN, with type I IFNs (α and β) highlighting as the most important, as they are associated with viral clearance. Type I IFN secretion can be induced via different pathways, activated by various components of the virus, such as surface proteins or genetic material. These molecules can trigger the activation of the IFN pathway trough surface receptors, including IFNAR, TLR4, or some intracellular receptors, such as TLR7, TLR9, and TLR3. Here, we discuss various types of dendritic cells found in humans and mice; their contribution to the activation of the antiviral response triggered by the secretion of IFN, through different routes of the induction for this important antiviral cytokine; and as to how DCs are involved in human infections that are considered highly frequent nowadays.

## Introduction

Dendritic cells (DCs) are known to be professional antigen-presenting cells (APC), as these cells are capable of presenting processed peptides from various antigens, initiating and modulating the adaptive immune response by activating both T and B lymphocytes (1, 2). Therefore, they are considered to be of great importance for the induction of an adequate adaptive immune responses (1, 3). DCs were first reported in 1973 by Steinman et al., where their morphological characteristics were defined, although it was not until 1998 that their function, as well as the proteins expressed on their surface, were characterized (1, 4). Since then, the knowledge acquired about DCs has increased over time.

During an infection, several molecules might be recognized and used to initially activate the innate immune system. These can be classified as pathogen-associated molecular or damage-associated patterns (PAMPs and DAMPs, respectively) (5, 6). These molecules will be recognized by pattern recognition receptors (PRRs) -expressed in most innate cells-, among which Toll-like receptors (TLRs), RIG-I-like receptors (RLRs), and NOD-like receptors (NLRs) are included. The interaction between PAMPs and PRRs promotes the release of cytokines, chemokines, and other chemical mediators that induce the inflammation of the infected tissue (7–9). TLRs are type I transmembrane glycoproteins that can be classified as extracellular or intracellular (10). Both TLR types have an N-terminal domain named leucine-rich repeat (LRR), a transmembrane domain, and a C-terminal cytosolic region named Toll/IL-1R (TIR). The main function of TLRs is to recognize a distinct set of ligands, such as proteins, genetic material, and carbohydrates (9–11). While ten different receptors of this type have been described up to date to be expressed by human cells, thirteen haven been described in mice (12, 13).

Identification of either PAMPs or DAMPs by immature DCs leads to the activation and consequent maturation of these cells (14, 15). This phenomenon is associated with changes in the phenotype and function of DCs, including the upregulation of costimulatory and adhesion molecules (16, 17). These cells are then capable of capturing antigens derived from pathogens, process, and present them to naïve T lymphocytes as peptides bound to class II major histocompatibility molecules (MHC) located on the surface of DCs. This structure is known as peptide-MHC complex and works as a cognate ligand for the T cell receptor (TCR), expressed by T lymphocytes (18–20). Antigen cross-presentation can allow the presentation of exogenous antigens on class I MHC molecules (21). Aside from this, the main function of MHC-I is to present antigens derived from the cytosol (22).

DCs are considered to be of great relevance in the clearance of viruses. Accordingly, significant amounts of cytokines, such as type I interferon (IFN), are required to achieve this clearance (2, 23, 24). In this review, we will discuss the classically and currently defined subsets of DCs, the importance of IFN in the antiviral response, and as to how DCs behave in some viral infections. This article aims to give insights into -and a better understanding of- the role played by DCs in the antiviral response toward important human viruses.

### Classically Defined Subsets of DCs and Their Phenotypes

Since their initial discovery, DCs have been deeply studied, and their immunophenotyping has allowed the definition of several subsets of DCs in the murine model, as well as in humans. Considering the immunobiology of the murine model, we highlight 2 important subgroups of DCs: conventional (cDCs) and plasmacytoid (pDCs). Both are derived from a common DC precursor (CDP), which is of myeloid origin (25, 26). Two precursor cell types arise from the CDP: a preDC and a pre-pDC. Moreover, the common lymphoid progenitor (CLP) has also been proposed to give rise to pDCs by numerous studies (27–29), suggesting that this particular cell type has a mixed ontogeny. Furthermore, murine cDCs are classified into two subsets: cDC1 and cDC2, which develop in the bone marrow (30). The cDC1 compartment comprises CD8α^+^ DCs and CD103^+^ DCs, while the cDC2 compartment comprises CD11b^+^ DCs (31). Recent findings regarding DC ontogeny are described in section Single-Cell RNA-seq Approach for the Characterization of Novel DCs Subtypes and Precise DC Ontogeny.

Human homologs of these subtypes have been discovered through various comparative phenotypic and functional analyses and, more recently, through genomic, transcriptomic, and proteomic analyses. Human CD141^+^ DCs are homologous to both CD8α^+^ murine DCs and CD103^+^ murine DCs, human CD1c^+^ DCs are homologous to murine CD11b^+^ DCs, and both humans and mice possess pDCs with similar functional characteristics (32). Classically defined DC sub-typing, their location, and commonly expressed surface markers can be found in Table 1. Functional and phenotypical characteristics of these cell types will be discussed in the following sections, and novel findings regarding DC subtypes will also be discussed.

**Table 1:** DCs subsets, their location, and their surface markers.

**DCs subset**		**Location**	**Surface markers**			**Reference**
cDCs	Mouse cDC1 (CD8α^+^)	Lymphoid organs	MHC-II^+^ Ly6C^−^ B220^−^ CD4^−^	CD8α^+^ CD11b^lo^ CD11c^hi^ CD24^hi^	CD205^+^ (DEC-205^+^) CD207^+^ (Langerin^+^) CLEC9A^+^ XCR1^+^	(33–36)
	Mouse cDC1 (CD103^+^)	Lymphoid organs and Non-lymphoid organs	MHC-II^+^ Ly6C^−^ B220^−^ CD8^−^ CD86^+^	CD80^+^ CD11b^+^ CD11c^+^ CD24^+^	CD103^+^ CD205^+^ (DEC-205^+^) CD207^+^ (Langerin^+^) XCR1^+^	(37–43)
	Human cDC1 (CD141^hi^)	Lymphoid organs and Non-lymphoid organs	CD11c^lo^ CD45^+^	CD141^hi^ (BDCA-3^hi^) CLEC9A^+^	XCR1^+^	(44–46)
	Mouse cDC2 (CD11b^+^)	Lymphoid organs and Non-lymphoid organs	MHC-II^+^ F4/80^−^ CD4^+^ CD11b^+^	CD11c^+^ CD24^+^ CD64^+^	CD103^−^ CD172a^hi^ (SIRPα^hi^) XCR1^lo/−^	(47, 48)
	Human cDC2 (CD1c^+^)	Lymphoid organs	MHC-II^+^ CD1a^lo/+^ CD1c^+^ (BDCA-1^+^) CD11c^+^	CD14^−^ CD16^−^ CD172a^+^ (SIRPα^+^)	XCR1^−^ FcεRI^+^ CD207^−^ (Langerin^−^)	(47, 49–53)
pDCs	Mouse pDCs	Blood, lymph nodes and lymphoid tissues	MHC-II^lo^ CD4^+^ CD11b^−^ B220^+^	CD11c^lo^ CD25^lo^ CD38^+^ CD40^−^	CD43^+^ CD62L^mid^ Ly6C^hi^	(54–56)
	Human pDCs	Blood and bone marrow	MHC-II^lo^ B220^+^ CD1a^−^ CD4^+^ CD11a^+^ CD11c^−^ CD13^−^	CD14^−^ CD16^−^ CD18^+^ CD33^−^ CD38^lo^ CD40^lo^ CD44^+^	CD54^+^ CD62L^+^ CD123^+^ (IL-3Rα^+^) CD127^−^ (IL-7Rα^−^) CD303^+^ (BDCA-2^+^) CD304^+^ (BDCA-4)	(57–60)

#### Murine DCs

Among the murine cDC1 subset, CD8α^+^ DCs are of particular importance. As their name suggests, this class of DCs is characterized by the expression of a CD8αα homodimer (distinct from the CD8^+^ T cell CD8αβ heterodimer) (37, 61), although no clear biological function has been attributed to it (62). CD8α^+^ DCs reside in both central and peripheral lymphoid organs, namely thymus, spleen, and lymph nodes (62–64). Some of the PRRs expressed by this subset are TLR3, TLR9, and TLR11/12 (65–68), and unlike other DC subtypes, CD8α^+^ DCs do not express TLR7 (68). These cells are capable of secreting large amounts of IL-12p70 upon activation (69), hence driving powerful T_H_1 responses when presenting antigens, and are specialized in cross-presentation of antigens on MHC-I molecules (70). These DCs are incapable of secreting interferon IFN-γ, although other DC subtypes do (69). Nonetheless, they are an important source of IFN-λ once they are activated via TLR3 (71). CD8α^+^ DCs are characterized by surface expression of CD205, langerin (CD207), C-type lectin receptor 9A (CLEC9A), and chemokine receptor XCR1 (33–36) Finally, murine CD8α^+^ DCs are also MHC-II^+^, CD4^−^, Ly6C^−^, CD11b^lo^, CD11c^hi^, CD24^hi^, and B220^−^.

Another cDC1 subset relevant for the understanding of viral pathogenesis is the CD103^+^ DCs compartment, characterized by the expression of the αE integrin CD103. These cells are present in many tissues, including the gut, lungs, spleen, skin, and various lymph nodes (38). CD103^+^ DCs have a developmental relation with CD8α^+^ DCs (72) and share characteristic surface markers like CD24, CD205, and langerin (39–42). However, CD103^+^ DCs are capable of inducing a more robust T_H_17 response through IL-1β and IL-6 secretion (73), and lack the expression of TLR3, although they remain responsive to polyI:C, a TLR3 agonist (74). Immunophenotyping has revealed an extensive heterogeneity among CD103^+^ DCs, although some common features among each compartment are the expression of different surface markers such as Ly6C^−^, CD11b^+^, CD11c^+^, CD80^+^, CD86^+^, B220^−^ and, of course, CD103^+^ (38). Most of them are CD8^−^, except for those that reside in the gut (namely, colon) (43) and spleen (37). Extensive murine subgroup immunophenotyping can be reviewed in del Rio et al. (38).

Importantly, CD103^+^ DCs have enhanced antigen cross-presentation capacities, which is employed by the immune system for the resolution of some viral infections, as well as for the maintenance of self-tolerance and tumor immune control, since phagosome-derived peptides can be presented on MHC-I molecules to activate -or inhibit- cytotoxic CD8^+^ T cells (75, 76). Regarding viral infections, skin, and lung CD103^+^ DCs subtypes have been shown to prime CD8^+^ T cells more efficiently than their CD103^−^ counterparts (77, 78), and those that are liver-resident can prime CD8^+^ T cells *in situ* (79). On the other hand, mediastinal lymph node (80) and splenic marginal zone (81) CD103^+^ DCs contribute to the maintenance of self-tolerance through cross-presentation of self-antigens, especially to CD4^+^ T cells (38, 82). As a matter of fact, CD103^+^ DCs have been shown to secrete TGF-β and induce the expression of the transcription factor Foxp3 on T cells, promoting the development of T_regs_ (80, 83, 84).

Even though the cDC2 subset has been less studied than the cDC1 compartment, their contribution to the activation of an immune response should be deeply characterized. These cells reside in both lymphoid and non-lymphoid tissues, such as lungs and guts. Unfortunately, their characterization has been hampered by their innate heterogeneity (85–88), the lack of various specific cDC2 markers, and their similarity with macrophages and other members of the phagocytic mononuclear system (89, 90). However, recent transcriptomic studies have circumvented these difficulties, making it possible to characterize adequately these cells due to their development routes (91). Contrary to cDC1 cells, cDC2 cells do not efficiently cross-present antigens. They can efficiently prime T_FH_ cells (92, 93) and tend to polarize adaptive immune responses toward T_H_17 and T_H_2 profiles (86, 94–96). In addition, cDC2s have been proven to be relevant mediators of asthma-like responses (94), hypersensitivity responses (94), and infections of bacteria (97, 98), fungi (86), and parasites (99, 100). Moreover, they have been regarded as important modulators of mucosal immunity, given their abundance in the gut and respiratory tract (47). This subset expresses particularly high levels of TLR4, TLR5, and TLR11 (18, 101, 102), which partly differentiates them from other DC subsets. Their hallmark marker is CD172a (SIRPα) and expresses virtually no XCR1, which are key characteristics for differentiating them from the cDC1 subset, (47). Phenotypically, these cells have been defined as F4/80^−^, MHC-II^+^, CD4^+^, CD11b^+^, CD11c^+^, CD24^+^, CD64^+^, CD103^−^, CD172a^hi^, and XCR1^lo/−^ (47), although lamina propria-residing intestinal cDC2s are CD103^+^ (48).

The last type of DCs we review are pDCs, which were first described in 2001 for mice (103) and in 1999 in human blood (57, 104). These cells are characterized both in mice and humans for their plasmacytoid appearance, their capacity to secrete large quantities of type I IFN (57, 103), and are mainly found circulating in the blood and secondary lymphoid organs (105–107), although they can migrate to inflamed skin (108), gut (109, 110), and epithelia (111). As mentioned above, pDCs arise not only from a myeloid precursor -the CDP- but also from a lymphoid precursor -the CLP-, giving this class of DCs a mixed ontogeny. Moreover, some murine pDCs may undergo conversion toward a cDCs phenotype (112, 113), which blurs the limit between myeloid and lymphoid DCs lineages even more. pDCs exhibit poor antigen presentation capabilities, are characterized by lower-than-average MHC-II levels in comparison to cDCs (54, 55), and have been found to aid in plasma cell differentiation through type I IFN and IL-6 secretion (114). Most notably, these cells represent a major source of IFN-α upon viral infections and express abundant TLR7 and TLR9 in their endosomal membranes (27). Regarding their pattern of expression of surface markers, the classical definition of pDCs involves medium-to-low levels of CD11c expression and high levels of B220, although such a definition may lead to confusion since it is too vague. Additionally, murine pDCs are CD4^+^, CD11b^−^, CD25^lo^, CD38^+^, CD40^−^, CD43^+^, CD62L^mid^, Ly6C^hi^, and, as previously stated, express lower MHC-II levels than cDCs (56).

#### Human DCs

In humans, CD141^hi^ DCs (BDCA-3^+^ DCs) represent the functional homolog of murine cDC1 cells (CD8α^+^ and CD103^+^ DCs), mainly due to their similar localization—both are found in lymphoid and non-lymphoid tissues, such as thymus and lymph nodes, show enhanced antigen cross-presentation to CD8^+^ T cells, T_H_1 response polarization, and extensive secretion of TNF-α and IFN-α/β/λ under TLR3 activation (44–46, 71, 115). Unexpectedly, a controversial study reported that CD141^hi^ DCs secrete neither IL-23p19 nor IL-12p70 in response to TLR3 activation by polyI:C or a cocktail of pro-inflammatory cytokines (45). Nonetheless, other reports suggest that CD141^hi^ DCs do indeed secrete IL-12p70 in response to TLR3 stimulation with polyI:C and other TLR3 agonists, further supporting the notion that these cells induce T_H_1 polarization (44, 115). CD141^hi^ DCs have been described as CD11c^lo^, CD45^+^, CD141^hi^ (BDCA-3^hi^), and vastly express XCR1, CLEC9A, and TLR3 (44–46), consistent with their cross-presentation capabilities and activation by soluble nucleic acids.

On the other hand, CD1c^+^ DCs (BDCA-1^+^ DCs) are the human homolog of CD11b^+^ murine DCs (cDC2s). These cells are present in blood, lungs, gut, skin, and lymphoid organs, such as the spleen, tonsils, and lymph nodes (88). In agreement with what has been observed in mice, human cDC2s express a wide variety of TLRs, such as TLR1, TLR2, TLR4, TLR5, TLR6, and TLR8 (116–118). Contrary to murine cDC2s, human CD1c^+^ DCs are more versatile when priming CD4^+^ T cells and can also polarize them toward a T_H_1 profile (117, 119), which is consistent with their ability to secrete high amounts of IL-12 (117, 120), and have been shown to efficiently drive T_FH_ responses (121). Moreover, they are also capable of cross-presentation of antigens to CD8^+^ T cells quite efficiently (120, 122). Thus, given their PRR repertoire and their cytokine secretion profile, CD1c^+^ DCs are excellent APCs in the context of either bacterial, viral, or fungal infections. Lastly, regarding their surface marker expression profile, these cells are characterized as MHC-II^+^, CD1a^lo/+^, CD1c^+^ (BDCA-1^+^), CD11c^+^, CD14^−^, CD16^−^, CD172a^+^ (SIRPα^+^), XCR1^−^, FcεRI^+^, and langerin^−^ (47, 49–52), although CD1a, CD1c and langerin expression is variable and/or inducible (50, 53).

**Figure 1:**
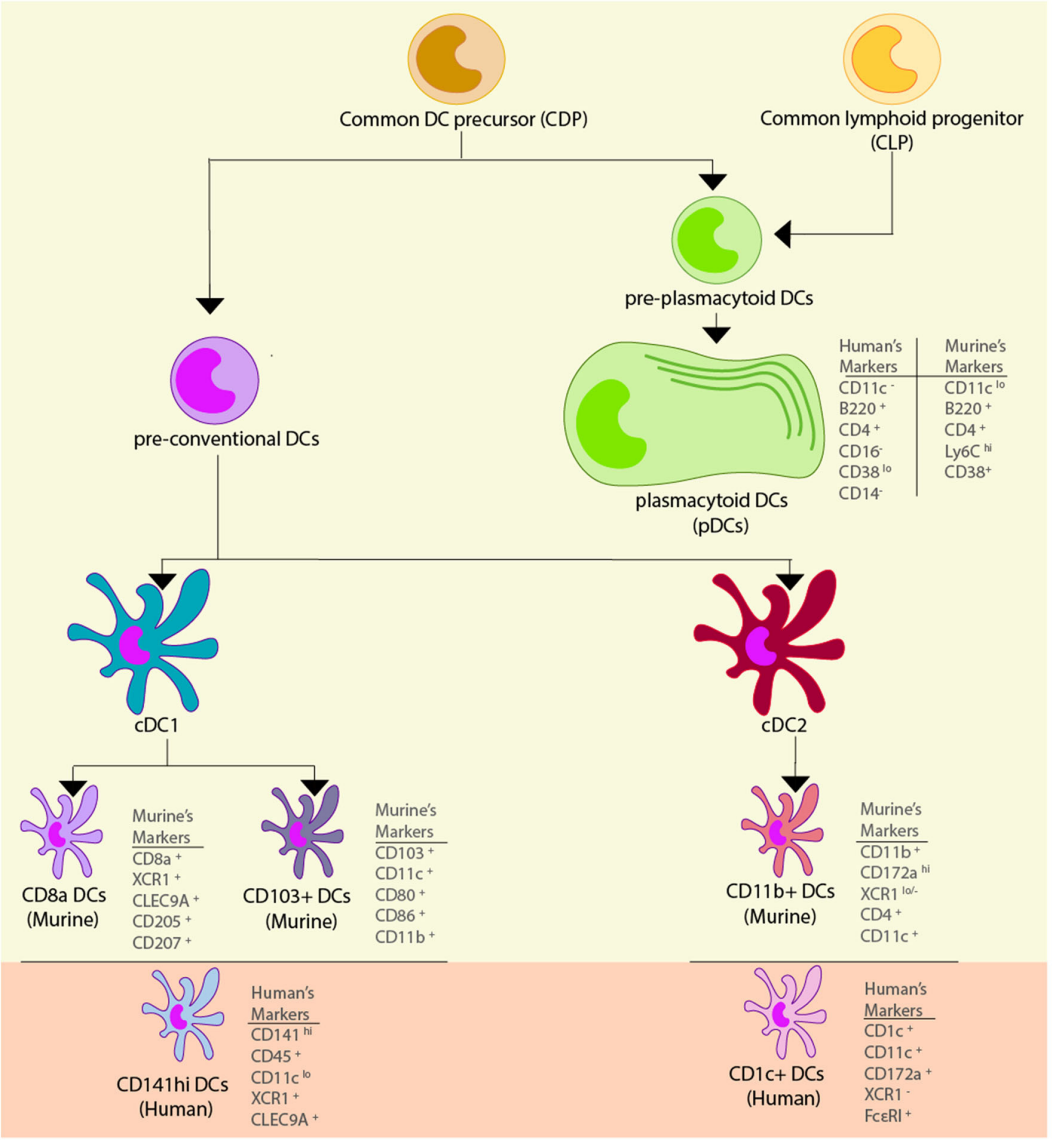
Dendritic cell subsets. Dendritic cells (DCs) are derived from a common myeloid precursor, from which two precursors can be developed. The first precursor corresponds to the pre-conventional DCs and in murine models they can become conventional DCs (cDCs) type 1 and 2, and within these cells there are different subtypes of DCs. In the cDC1 found two subtypes of cells can be found: CD8^+^ DCs which has the markers CD8^+^, XCR1^+^, CLEC9A^+^, CD205^+^, and CD207^+^, and CD103^+^ DCs that has the markers CD103^+^, CD11c^+^, CD80^+^, CD86^+^, and CD11b^+^. The functional homolog of these cells in human are the CD141^hi^ DCs, and their markers correspond to CD141^hi^, CD45^+^, CD11c^lo^, XCR1^+^, and CLEC9A^+^. In the cDC2 subsets are comprised the CD11b^+^ DCs which has the markers CD11b^+^, CD172a^hi^, XCR1^lo/−^, CD4^+^, and CD11c^+^. The functional homolog of these cells in human are the CD1c^+^ DCs, and their markers are CD1c^+^, CD11c^+^, CD172a^+^, XCR1^−^, and FcεRI^+^. The second precursor corresponds to the pre-plasmacytoid DCs that can become plasmacytoid DCs (pDCs), and in murine models its markers correspond to CD11^lo^, B220^+^, CD4^+^, Ly6C^hi^, and CD38^+^, while in human, their markers correspond to CD11^−^, B220^+^, CD4^+^, CD11a^+^, and CD38^lo^.

Finally, human pDCs (BDCA-2^+^ DCs) share many of their characteristics with murine pDCs: they are found mainly in blood, secrete high amount of IFN-α upon activation (123, 124), and share some but not all immunophenotypic characteristics since human pDCs are CD4^+^ and B220^+^, but pDCs additionally have CD11c^−^, CD14^−^ and CD16^−^ as surface markers (58). It is important to note that pDCs' repertoire of PRRs is very specialized and consists almost exclusively of TLR7 and TLR9 (58, 123, 125). Because of their enhanced type I IFN secretion and nucleic acid-oriented sensing of pathogens through endosomal TLRs, pDCs exhibit vast antiviral activities and are only mildly permissive to viral infections compared to cDCs (126, 127). Moreover, they express lower-than-average levels of MHC-II and are CD1a^−^, CD11a^+^, CD13^−^, CD18^+^, CD33^−^, CD38^lo^, CD40^lo^, CD44^+^, CD54^+^, CD62L^+^, CD123^+^ (IL-3Rα^+^), CD127^−^ (IL-7Rα^−^), CD303^+^ (BDCA-2^+^), and CD304^+^ (BDCA-4^+^) (57, 59, 60) The different surface markers, and specific characteristics associated with each dendritic cell type describe above are shown in Figure 1 and in Table 1.

### Single-Cell RNA-Seq Approach for the Characterization of Novel DCs Subtypes and Precise DC Ontogeny

Even though the classical definition of DCs subtypes involves two classes of cDCs and one class of pDCs, the recent development and more widespread application of single-cell RNA sequencing (scRNA-seq) and cytometry by time-of-flight (CyTOF), have shown that DC subsets can be far more diverse than previously thought. While CyTOF has shown to be useful for the thorough characterization and definition of DC subtypes based on the expression of surface markers, scRNA-seq provides a useful and versatile technique that has allowed to define previously overlooked DC subsets and trace their ontogeny, as well as of other hematopoietic progenitors using transcriptomic data analyses. These approaches have allowed to further characterize several features for each subclass (91, 128–131).

For instance, the transcriptomic changes associated with LPS exposure in splenic DCs by scRNA-seq was recently characterized (132). Not only did they find significant differences in gene expression among cDC1s, cDC2s, and pDCs before and after LPS exposure, but they also found significant and inherent diversity among the classically defined cDC2 subset (132). Thus, they were able to identify 4 classes of cDCs, as well as one class of pDCs, by the analysis of transcriptional profile for each cell (132).

Three years later, Villani et al. found 6 classes of DCs in human peripheral blood by FACS sorting, followed by scRNA-seq and named them DC1-6 accordingly (133). The DC1 cluster corresponded to classically defined cDC1s (133). The DC2 and DC3 clusters corresponded to two transcriptomic profiles found to be distinct among classically defined cDC2s, and the DC3 cluster was found to possess an inflammatory gene expression signature (133). The finding of two distinct subclasses of cDC2s is not surprising, considering the evidence of functional and transcriptional heterogeneity among this group (130, 132, 134). The DC4 cluster corresponded to an MHC-II^+^, CD11c^+^, CD1c^−^, CD141^−^ population, that did not meet neither cDC1, cDC2, nor pDC inclusion criteria (133). The DC5 cluster was unprecedented and corresponded to MHC-II^+^, CD11c^−^, CD123^−/+^ DCs. This cluster was characterized by a high expression of *AXL* and *SIGLEC6* and was named AS DCs (133). These cells were found to be in close relation to pDCs (DC6, the last cluster), since they expressed a few similar markers, such as CD123 and CD303. However, they were functionally distinct, since they did not produce IFN-α upon stimulation. Moreover, they secreted IL-12p70 and IL-8, upregulated CD86 upon activation, and were able to prime T cells, whereas sorted “pure” pDCs -gated excluding AS DCs- did not (133). This is quite remarkable, as previous studies show IL-12p70 secretion, CD80 upregulation, and differences in the expression of the pDC hallmark transcription factor E2-2 among pDCs (135–137), a result that could be biased because of the lack of discrimination between pDCs and the newly discovered AS DC subset. Moreover, they could explain some of the heterogeneity observed in this subset (137–139).

The existence of a new class of cDCs (cDC3) was proposed both mice and humans based on their transcriptomic fingerprint using scRNA-seq (140). Interestingly, this new type of DC was discovered in lung tumors of patients and were also found in murine lung carcinomas. Although these cells shared many features with cDC1s, they lacked the expression of key markers, such as XCR1 and CLEC9A and clustered as an independent group (140). Moreover, cDC3s expressed high levels of transcripts associated with an LPS-activated state defined in a previous study (141). The authors did not find the AS DC subset (DC5 cluster) previosuly described in tumor samples (140). Reciprocally, this last study did not find the cDC3 subset described in blood samples (140). Thes sudies have suggested that this is likely attributed to differences in DC states between blood samples from healthy patients and the tumor environment they analyzed (140).

Lastly, three phenotypic variants for human pDCs where defined after stimulation with the influenza virus, which were named P1, P2, and P3 (142). The definition of these subsets was made based on the expression of CD80 and PD-L1. Different morphologies, as well as distinct surface markers and transcriptomic fingerprints between subsets, were observed (142). Moreover, only cells from the P1 subset (CD80^−^, PD-L1^+^) were able to secrete IFN-α in response to virus stimulation and cells of the P3 (CD80^+^, PD-L1^−^) subset had higher migration and CD4^+^ T cell expansion (142). What is interesting about this study is that their gating strategy allowed the sorting of pDCs, while avoiding AS DCs and pre-DC contamination, as previously reported (142). These findings indicate that the observed effects are due to *bona fide* pDC subsets, rather than pDC contamination with other DC subsets (142).

scRNA-seq has also been used to elucidate the intricate network that represents myeloid stem cell differentiation (143–145), which has shown to be particularly useful in the field of DC ontogeny. Classically defined human cDC1, cDC2, and pDC transcriptomic phenotypes are imprinted during ontogeny, rather than by environmental cues in DCs found in blood and secondary lymphoid organs Further, recent studies strongly support the notion that myeloid common DC precursor can give rise to pDC and pre-DC precursors, from which arise pre-cDC1 and pre-cDC2 progenitors, each committed to maturation toward cDC1 and cDC2, respectively (49, 146, 147). Common upregulated and downregulated genes between pre-cDC1 and pre-cDC2, as well as pre-cDC1- and pre-cDC2-specific upregulated genes, and their temporal onset of expression have been determined in mice (147). Moreover, SiglecH and Ly6C can serve as lineage markers during DC development and lineage commitment among pre-DCs occurs in the bone marrow in mice -SiglecH^+^ precursors develop into SiglecH^−^ pre-DC precursors, which in turn can be cDC1-committed pre-cDC1s if they are Ly6C^−^, or cDC2-committed pre-cDC2s if they are Ly6C^+^ (147).

Altogether, these results suggest that we should rethink the current classification of DCs and then evaluate a more thorough definition of DC subtypes in future studies, considering the possible contamination between subsets that could occur [e.g., pre-DCs and pDCs in mice (146), or AS DCs and pDCs in humans (133)]. Undoubtedly, scRNA-seq, as well as CyTOF, are powerful tools that will continue to unravel the immunobiology of DCs.

## Importance of Interferon Regulation for the Immune Response Against Viral Infections

IFN is a characteristic antiviral cytokine able to modulate the innate and adaptive immune response (148). This cytokine was discovered in the 1950s (149) and is divided into three different groups: Type I, II, and III IFN, whose function is regulated by the JAK-STAT pathway (150). In humans, type I IFN is divided into IFN-α, IFN-β, IFN-ε, IFN-κ, IFN-ω; type II into IFN-γ; and type III into IFN-λ, which is divided in IFN-λ1, IFN-λ2, IFN-λ3, IFN-λ4 (151, 152). Similarly to humans, in mice, IFNs are divided into three groups—type I IFN includes IFN-α, IFN-β, IFN-ε, IFN-κ (153, 154), type II comprises IFN-γ, and type III considers IFN-λ.

**Figure 2:**
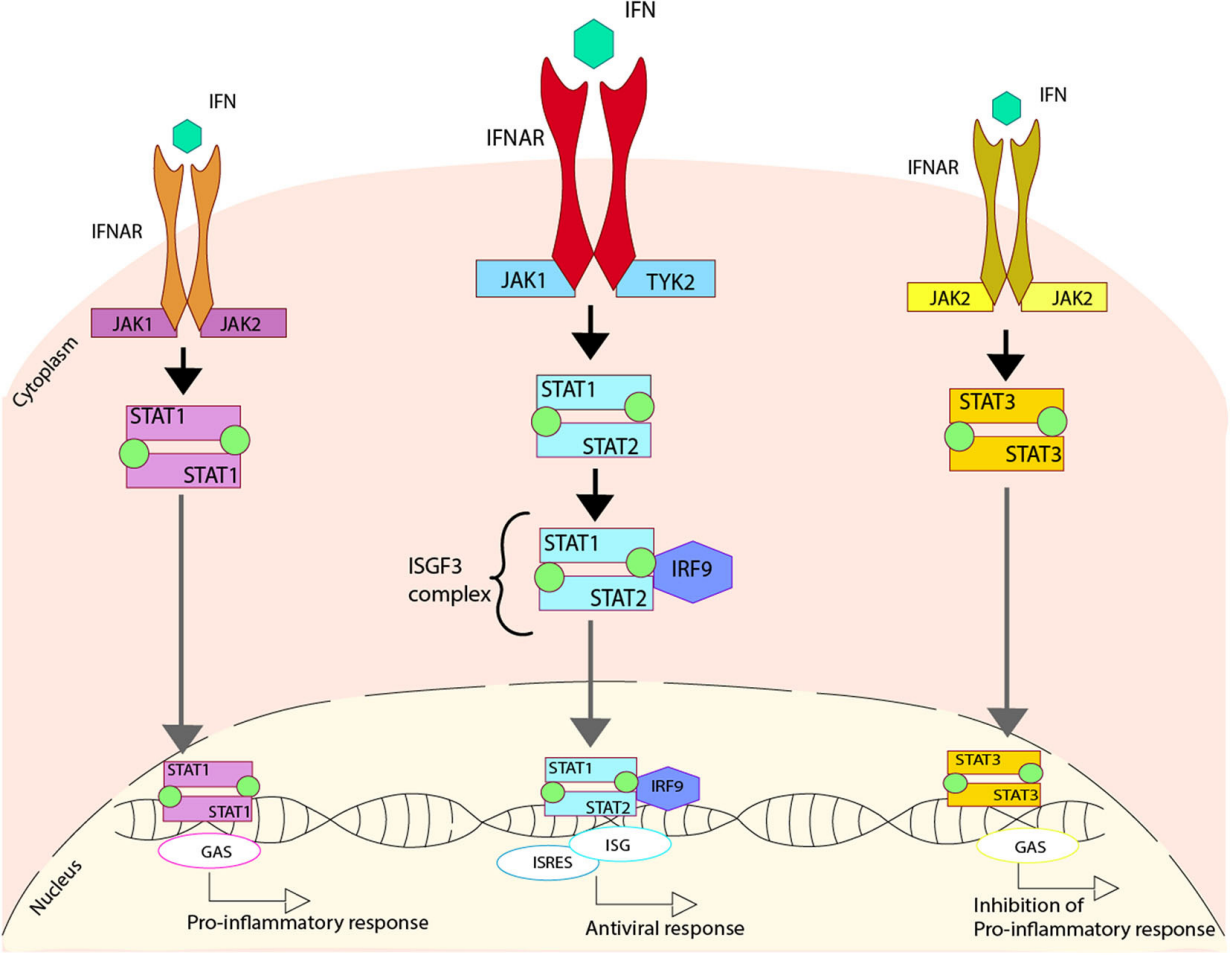
Regulation of type I IFN due to the activation of IFN receptors. Upon activation of the IFNAR receptor induced by the cytokine IFN, three different pathways can be activated. One pathway involves the phosphorylation of the IFNAR cause by Janus Kinase 1 (JAK1) and Tyrosine Kinase 2 (TYK2), which generates the phosphorylation of both STAT1 and STAT2 that come together to form a heterodimer. This heterodimer interacts with IRF9, forming the ISGF3 complex that is translocated to the nucleus where it binds to DNA activating the regions of ISG and ISRES, promoting an antiviral response. An alternative pathway involves the phosphorylation of the IFNAR caused by JAK1 and JAK2, which produces the phosphorylation of STAT1 and two of them come together in order to form a homodimer. This homodimer is able to translocate to the nucleus where it binds to DNA, activating the regions of GAS, and promoting a pro-inflammatory response. Another alternative pathway involves the phosphorylation of the IFNAR, caused by JAK2, which generates the phosphorylation of STAT3, and two of these come together in order to form a homodimer. This homodimer is able to translocate to the nucleus where it binds to DNA, activating the regions of GAS and promoting the inhibition of the pro-inflammatory response.

Type I IFN is secreted in response to viral infections when PRRs identify viral proteins or genetic materials, triggering the IFN secretion (155, 156). The IFN secreted is later recognized by the IFNAR receptor into the surface of infected cells, promoting the activation of the JAK-STAT pathway. The activation of these pathways starts with the phosphorylation of IFNAR by two enzymes, Janus Kinase 1 (JAK1) and Tyrosine Kinase 2 (TYK2). This phosphorylation modulates the activation of both STAT1 and STAT2, through their phosphorylation and later heterodimerization. This phosphorylated STAT1/STAT2 complex interacts with IRF9 to make the ISGF3 complex that is translocated to the nucleus. Once this complex is in the nucleus, it binds to the DNA sequence where it can activate the IFN-stimulated response elements (ISREs), some antiviral genes and the expression of IFN stimulated genes (ISGs), which are essential to promote the antiviral response in the host (157–161) (Figure 2).

Additionally, two other pathways can be activated when IFN is recognized by IFNAR. The first is triggered by the homodimerization of the phosphorylated STAT1 that is translocated into the nucleus and recognizes the gamma-activated sequence (GAS) within the DNA, activating a pro-inflammatory response (162). The other pathway is triggered by the homodimerization of the phosphorylated STAT3, which is translocated to the nucleus, where it is also able to recognize GAS. This interaction promotes the inhibition of the pro-inflammatory response, probably by an unknown self-control system able to sense the damage (163, 164) (Figure 2).

**Figure 3:**
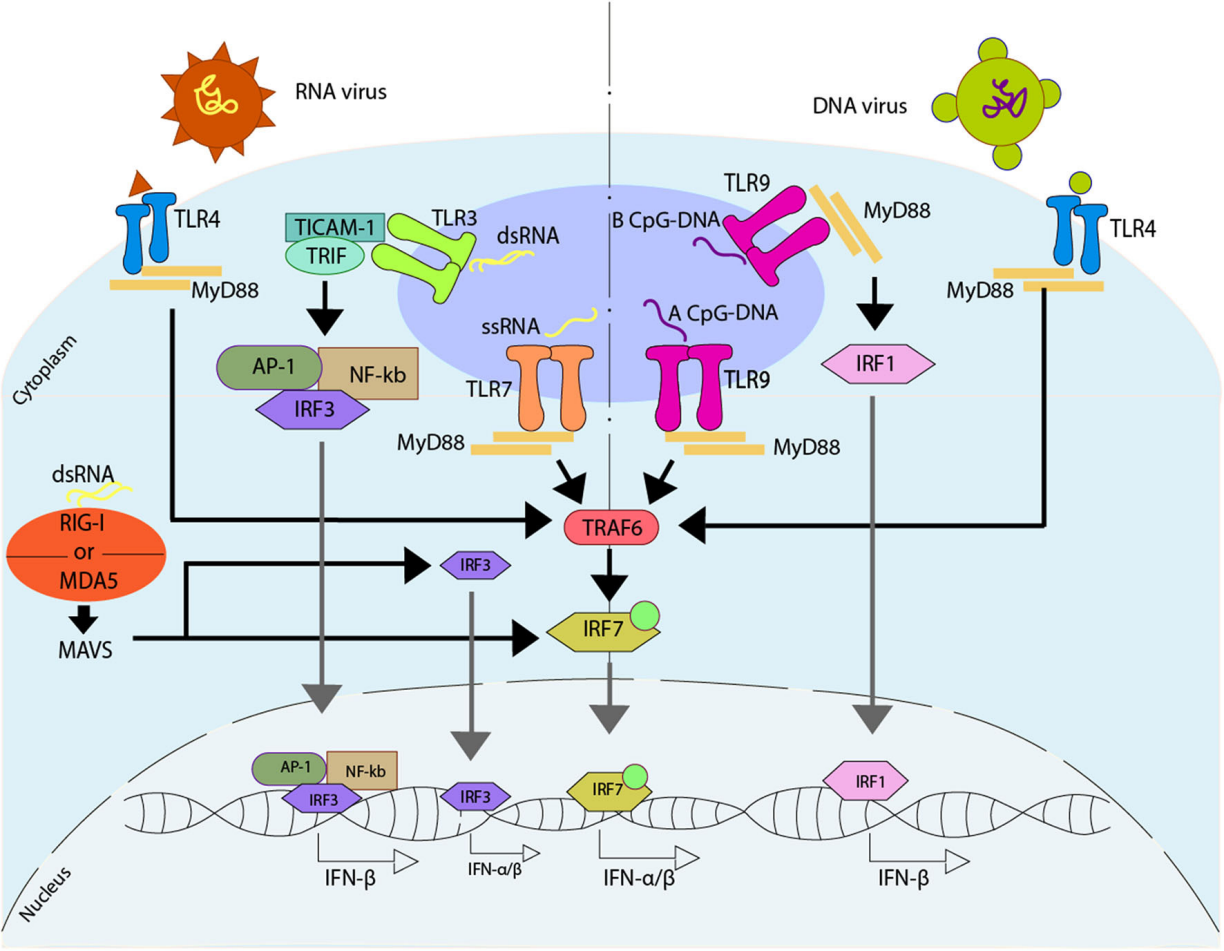
Regulation of type I IFN due to the activation of Toll-like receptors. Upon activation of Toll-like receptor (TLR) induced directly by viruses, different pathways can be activated. TLR7 recognize ssRNA, promoting the binding with MYD88, that will later interact with TRAF6, activating IRF7 which is translocated to the nucleus where it binds to DNA, promoting the release of IFN-α/β. TLR3 recognize dsRNA, promoting the interaction with TICAM-1 and TRIF, that activates AP-1, NF-κB, and IRF3, which are translocated to the nucleus, where they bind to DNA, promoting the release of IFN-β. The dsRNA can activate an alternative pathway, which can be sensed by either retinoic acid-inducible gene I (RIG-I) or by melanoma differentiation-associated protein 5 (MDA5). These receptors activate mitochondrial antiviral-signaling protein (MAVS), and by doing so they promote the IRF3 and/or IRF7 activation, and later translocation into the nucleus where they bind to DNA, promoting the release of IFN-α/β. There are two ways of activating TLR9, one is through the recognition of a type A CpG-DNA, which activates the pathway mediated by MYD88, similar to TLR7, while the other one involves the recognition of a type B CpG-DNA, where TLR9 binds with MYD88, activating IRF1, which is then translocated into the nucleus where it binds to DNA promoting the release of IFN-β. On the other hand, TLR4 is able to recognize viral peptides, activating a MYD88 -depending pathway similar to TLR7.

Another pathway described to promote an antiviral immune response associated with type I IFN response is through the activation of different Toll-like receptors (TLR) (165–167). TLR7 is activated by guanosine/uridine-rich ssRNA from viruses (168–170), while TLR9 is characterized by the recognition of methylated CpG rich DNA (171). However, both TLR7 and TLR9 trigger a common pathway mediated by MYD88 (167). Specifically, TLR9 can promote IFN-α or IFN-β, depending on the type of dendritic cells and their stimuli. In plasmacytoid DCs (pDCs), TLR9 recognizes type A CpG oligonucleotides (CpGA) promoting the binding of MYD88 that later interacts with TRAF6 triggering IRF7 activation. This activation of IRF7 is dependent on the ubiquitin E3 ligase activity of TRAF6. Once IRF7 is activated, it can phosphorylate independently of TBK1/IKK, and it can be translocated into the nucleus to promote the activation of the type I IFN-α/β (172– 175). Additionally, TLR9 can recognize type B CpG oligonucleotides (CpGB) in pDCs, stimulating the maturation of this cell type but inducing low levels of IFN-α (176). On the other hand, in cDCs and other cell types -such as macrophages-, TLR9 recognizes CpGB promoting the binding of MYD88 and the later interaction with IRF1, which is translocated into the nucleus to promote the activation of the only type I IFN-β (173). This pathway, unlike the one described above for pDCs, does not require the use of IRF3 or IRF7 (173) (Figure 3).

Another typical TLR associated with the antiviral response is TLR3, which is found in endosomes of cDCs and is capable of recognizing dsRNA (177). Once it identifies the dsRNA, TLR3 interacts with the adaptor protein TIR-containing adaptor molecule-1 (TICAM-1/TRIF). This interaction promotes the activation of AP-1, IRF3, and NF-κB, which then are translocated into the nucleus to promote the expression of IFN-β. Additionally, the activation of the NF-κB pathway allows the expression of pro-inflammatory genes (178–180).

Finally, TLR4, who is localized in the surface of cells, is able to identify different viral proteins, triggering the signaling and activation of type I IFN genes through the MYD88 pathway (181). Additionally, TLR4 can modulate the activation of type I IFN genes through other ways associated with TRIF, where TLR4 interacts with TRIF through TRAM, triggering a late activation of the MYD88 pathway, and the expression of type I IFN genes against viral infection (182, 183) (Figure 3). Similar to TLR4, TLR2 -which is also localized in the surface of the cells- has also been associated with the identification of viral proteins (184).

## Antiviral Activity of DCs Against RNA Viruses

As was mentioned above, DCs subsets are crucial players in the host defense against pathogens, particularly viruses. In this section, we will discuss the antiviral role of DCs against RNA viruses that we consider epidemiologically relevant and elicit a well-described antiviral activity of DCs, such as the human respiratory syncytial virus, the human metapneumovirus, Influenza virus, the hepatitis C virus, and the human immunodeficiency virus. The different PAMPs from each virus are mentioned in Table 2.

**Table 2:** Viruses and some of their PAMPs.

**Type of virus**	**Virus**	**PAMPs**	**References**
RNA genome	Human respiratory syncytial virus	(-)ssRNA Nucleoprotein (N)	(185–187)
	Human metapneumovirus	(-)ssRNA Glycoprotein (G)	(188–190)
	Influenza virus	(-)ssRNA Hemagglutinin (HA) Neuraminidase (NA)	(191–193)
	Hepatitis C virus	(+)ssRNA Non-structural (NS)3 Non-structural (NS)4A Non-structural (NS)5	(194–197)
	Human immunodeficiency virus	(+)ssRNA	(198, 199)
DNA genome	Papillomavirus	dsDNA E6 protein E7 protein	(200–203)
	Adenovirus	DNA	(204)
	Hepatitis B virus	DNA	(205, 206)
	Human alphaherpvirus	DNA US11 proteins	(205–207)
	Human alphaherpesvirus 3	DNA	(208)
	Human gammaherspesvirus 4	dsDNA	(209)

### Human Respiratory Syncytial Virus

Human respiratory syncytial virus (hRSV) is an enveloped member of the *Pneumoviridae* family, which has a single-stranded negative-sensed RNA genome (185, 186). The disease caused by hRSV is characterized mainly by the infiltration of eosinophils and neutrophils into the airways. An increase infiltration of neutrophils can contribute to lung damage (210–212). Even though CD8^+^ T cells are key in the clearance of the virus, it has been reported that the depletion of the CD4^+^ and CD8^+^ T cells in mice decreased the severity of the illness (213–215).

As expected, the antiviral immune response against hRSV is commanded by DCs. The different subsets of DCs in the lung play a specific role in the antiviral immune response against this virus. The pDCs are susceptible to hRSV infection in humans (127) and mice (216), increasing the expression of molecular surface markers such as CD80, CD86, and CD40, indicating the maturation of these cells (217). Interestingly, a report indicated that upon infection with hRSV, the amount of pDCs was less in preterm infants as compared to those born at term (218). Studies in human monocyte-derived DCs (moDCs) and pDCs showed that both subsets of DCs could be infected by hRSV, and their infection promotes the secretion of several cytokines such as IL-6, IL-10, TNF-α, IL-1β, and IL-12p70 (24). One of the differences of the effect of hRSV infection is that the induction of IFN-α was detected only in pDCs, and this infection can inhibit the production of type I IFN in cDCs and pDCs through the recognition of TLRs agonist (24, 219). However, the absence of pDCs does not alter the levels of IFN-α during the infection with hRSV (220).

In a murine model, Smit et al. described that the number of cDCs and pDCs were increased in the lung and also, in the lung-draining lymph nodes of BALB/c hRSV-infected mice during the acute phase of the infection (221). Moreover, when pDCs were depleted, the immunopathology caused by hRSV was enhanced in the lung of infected mice, as seen by an increase in the viral loads. This result demonstrated that pDCs are relevant during hRSV clearance (221). According to this, in the absence of pDCs, the levels of IFN-α were drastically decreased, consistently with previous reports (216, 221, 222).

Moreover, it has been described that hRSV infection of cDCs does not alter the expression of maturation surface markers in adult mice. However, in neonates, the CD80 and CD86 maturation markers are decreased (223). These findings are in accordance with the severity of the immunopathology described in neonatal and adults mice models (223). As APCs, cDCs are important in the activation of T cells, but this essential function is altered by hRSV infection (224). Interestingly, hRSV infection on cDCs impairs the immunological synapses mediated by the surface expression of the hRSV nucleoprotein (187, 224). This impairment can explain why the host displays an inefficient immune response against hRSV and why the host can be re-infected by this virus throughout its life.

### hMPV

The human metapneumovirus (hMPV) also belongs to the *Pneumoviridae* family, and it has a single-stranded RNA genome with a negative sense (188). This virus is globally recognized as the second most important agent that causes bronchiolitis and pneumonia in susceptible individuals (225). Noteworthily, the mechanisms by which hMPV can evade the establishment of an appropriate immune response are still poorly characterized in humans, but recent studies in BALB/c mice suggest that infiltrating T cells play a pathogenic role during hMPV infection (226, 227).

It has been described that DCs are susceptible to be infected by hMPV and, depending on the strain and the host, the replication in these cells can be productive or abortive in an *in vitro* model of human cDCs (228) or mouse bone-marrow-derived DCs (BMDCs) (229), respectively. Moreover, hMPV can infect both human moDCs and pDCs, which promote the maturation of the cells (24). Furthermore, in hMPV-infected DCs, the production of IFN-α is increased, but this effect is inhibited in response to TLR agonists (24). Compared to hRSV, hMPV is more susceptible to the antiviral effect of IFN-α. This observation can be explained by the lack of NS1 and NS2 proteins in hMPV, present in hRSV, that have been described to interfere with the type I IFN signaling (230).

The infection of DCs by hMPV also impairs the ability of these cells to establish an efficient immunological synapse that allows T cell activation (229). One of the most important virulence factors of hMPV is the G glycoprotein (189, 190, 231). This protein has a role in the inhibition of the TLR4-depending signaling in moDCs. Kolli et al. demonstrate that human hMPV-infected moDCs downregulate the expression of the TLR4. Moreover, when evaluating DCs obtained from a mouse with a silenced TLR4 and infected with hMPV, the cytokines induced by the infection, such as IL-6, IL-10, CCL5, and IFN-β, were also decreased (190). Besides, to demonstrate the role of the G-hMPV glycoprotein, the authors tested a recombinant hMPV-ΔG virus. The data showed that, in the absence of the G-hMPV glycoprotein, there was increased production of cytokines and a moderate change in the IFN type I expression (190, 231). According to these results, TLR4 plays an important role in the activation of DCs during an hMPV infection.

### Influenza Virus

Influenza virus (IV) belongs to the *Orthomyxoviradae* family, with a negative-sensed, single-stranded, and segmented RNA genome (191, 192). This virus can be classified into types A, B, C, and D, being the influenza A virus (IAV), the most prevalent and responsible for the pandemics (192, 232). In the viral surface, the major glycoproteins are the hemagglutinin (HA) and neuraminidase (NA), being these proteins the basis of the classification of the IVs subtypes such as H1N1, H2N3, and H7N9, among others (193). As hRSV and hMPV, IAV also infects humans (233) and murine (234) DCs, although this infection is lower in murine DCs, and its infectivity depends on the type of HA protein in the virus (234). In the case of human DCs, the cDCs are more susceptible to the IAV infection than pDCs, and, in this context, the IAV-infected cDCs are less efficient to activate CD8^+^ T cells (233).

During IAV infection, DCs are one of the most relevant cell types in the initiation of the host immune response. It has been described that the recombinant HA proteins from A/WSN/33 (H1N1) and A/Thailand/KAN-1/2004 (H5N1) can induce cDCs maturation, and therefore the production of cytokines such as TNF-α and IL-12p70 (235). Also, TLRs on DCs are relevant for the recognition of IAV infection. The cDCs' activation occurs via TLR3 and TLR9 signaling (235, 236), whereas the activation of pDCs is via TLR7 (237) (Figure 3).

### Hepatitis C Virus

Hepatitis C virus (HCV) is part of the *Flaviviruses* family and presents a positive-sensed, single-stranded RNA genome (194). The prevalence of HCV represents a 2.2% infected subjects worldwide, and it is known for causing hepatitis to a chronic level that can even lead to hepatocellular carcinoma (195, 238, 239). Two potential antiviral targets are the nonstructural (NS) protein 3, which has a helicase and protease activity, and NS5, which has an RNA-dependent RNA polymerase activity. However, the peculiarity of this virus allows it to mutate frequently and generates up to six different genotypes (195). Even though hepatocytes are its principal target cell, it has been found that this virus can infect DCs at a minor level, interfering with its capacity to stimulate allogeneic T cells and to secrete IFN-γ (238, 240, 241).

HCV can evade the antiviral response due to the activity of the NS3/4A protease, which can inhibit the RIG-1 pathway, and the pathway where the TRIF protein participates (196, 197). It has been reported that when HCV infects hepatocytes, it can inhibit type I IFN secretion. However, evidence suggests that there are other cells in the liver capable of stimulating the ISG, during infection with this virus (242–244). Within a liver infected by HCV, pDCs are found in great numbers, even though the infection with this virus can decrease the number of pDCs and inhibit their function (245–247). It seems that through cell-to-cell contact between the cells infected with HCV and pDCs, the latter can secrete type I IFN as a result of the signal produce by TLR7, due to the sensing of the RNA of the virus, without infecting pDCs directly (243).

### Human Immunodeficiency Virus

The human immunodeficiency virus (HIV) belongs to the *Retroviridae* family and has two positive-sensed and single-stranded RNA molecules (198, 199). This virus is divided into two types, HIV-1 and HIV-2, where HIV-1 is the one described as a pandemic affecting ~37 million people (248–250). The progression of the disease due to the continuous activation of the immune system and the low levels of CD4^+^ T cells can lead to the development of the acquired immunodeficiency syndrome (AIDS) (251, 252). Studies *in vitro* with human DCs have demonstrated that some of these cells are susceptible to the infection with HIV, such as Langerhans cells, cDCs, and pDCs (253, 254). Interestedly, cDCs from HIV patients have shown low levels in the blood, and their function is compromised (255). When HIV infects DCs, it can travel within the cell until it locates memory CD4^+^ T cells into the lymphoid tissue, spreading the infection in this way (256, 257). Langerhans cells are the first cells that interact with HIV, and studies in these types of cells have demonstrated that Langerin receptors can block the transmission of the virus, making it possible to avoid the infection (258). Upon the HIV-infection of cDCs, the anti-viral pathways within these cells are changed, and as a result, it promotes the viral spreading. However, the use of antiretroviral therapies (ART) can return completely the functionality of the pathways IL-1 and type I IFN (255, 259).

The HIV ssRNA is recognized by TLR7, stimulating the MYD88 pathway to activate IRF7 in pDCs (260, 261). The secretion of IFN-α promotes the expression of the HIV restriction factor through the stimulation of IFNAR, thus inducing the activation of the JAK1/TYK2 pathway (262). The activation of pDCs is desirable to stimulate the production of IFN-α/β since it can control the spreading of the virus, but the continuous secretion of this cytokine can cause more damage than benefit (263, 264). The negative effect of a continuous induction of type I IFN involves the chronic activation of the immune system, immunosuppressive pathways are activated, and immunopathogenesis, along with immunodeficient syndromes are detected (264). Even though pDCs are no the main source of IFNs during HIV-infection, these cells play an important role during the infection due to the capability to target infected-cells and, at the same time, promote the immunopathogenesis (265). Even more, as a consequence of the constant activation of pDCs and the immune system, the chances of developing AIDS are increased (266, 267). There is an alternative pathway by which TLR7 is able to activate NF-κB, which can trigger the production of type I IFN as well (261, 268). Even though pDCs have two different pathways that promote the secretion of type I IFN, the amount of IFN secreted is lower compared to LPS-stimulated DCs (269).

## Antiviral Activity of DCs Against DNA Viruses

The role of DCs during DNA virus infections have also been characterized -thoroughly or partially, depending on the pathogen. In this line, infections and diseases caused by Papillomavirus, Adenovirus, hepatitis B virus, and Herpesvirus will be discussed in the following section, as DNA viruses that are considered to be epidemic viruses. Accordingly, these viruses have been described as epidemiologically relevant, since they cause several diseases with high incidences worldwide. The different PAMPs from each virus are mentioned in Table 2.

### Papillomavirus

Human papillomavirus (HPV) is a dsDNA virus recognized as the main etiological agent of cervical cancer, and it is also associated with carcinomas of the vulva, vagina, anus, and penis (200–202). However, infection with this virus can lead to several pathologies, such as cutaneous warts, squamous intraepithelial lesions, and even respiratory papillomatosis (200, 201). In this line, HPV exhibits a tropism for cutaneous and mucosal epithelial cells -without inducing the destruction of the infected cells-, thus explaining the type of carcinomas that it can cause (200). Remarkably, there are over 200 different species of HPV, divided into five different groups -alpha, beta, gamma, mu, and nu- and they are categorized according to their tropism and genetic composition (202, 270). The alpha group is the most studied and characterized so far, with HPV species in this subset dived as high or low risk (201).

Since immune surveillance is crucial for the establishment of persistence and the appearance of skins lesions, and epithelial cells -particularly keratinocytes- are the main target of infection for this virus, the DCs subtype that will play a more critical role in the regulation of this infection are Langerhans cells (LCs) (271–273). Remarkably, infection of LCs with HPV results in no expression of the genes of this virus, therefore being non-productive (271, 272). In light of this, cross-presentation performed by LCs is a fundamental step for the activation of T cells, once the former reaches the LN, during infection with HPV (274). A down-regulation of MHC-I has been reported during cervical carcinomas in human studies, which has also been associated with HPV-related carcinoma (275). This suggests that there is a decrease in the achievement of a proper T cell activation in this HPV-associated cancer, which, along with its immune evasion-associated capacities, makes this virus more virulent.

Upon infection, keratinocytes will secrete several pro-inflammatory cytokines that will induce the recruitment and activation of LCs (276–278). Recognition of HPV by DCs will induce their activation and eventual maturation (271–273). However, keratinocytes have also been reported to secrete anti-inflammatory molecules -such as TGF-β and IL-10- upon infection with HPV and once the tumor has been established, therefore down-modulating the activation of LCs (277). This results in an increased capacity for immune evasion for this virus. In this line, it has also been described that TLR3, TLR5, TLR8, and TLR9 pathways are activated during HPV infections (203, 278–280). However, two proteins of HPV (E6 and E7) have been described to down-modulate TLR9 expression -and IFN synthesis along with it-, once again favoring its immune evasion capabilities (203). Remarkably, overexpression of TLR4 has been described in HPV-infected cells, and this is correlated with resistance to apoptosis in these infected cells (280).

DCs -particularly LCs- will also play a significant role during cervical cancer that may induce HPV (272). As expected, immature LCs will sense their environment, capture and process antigens, and migrate to their respective LN. However, several studies have shown that, during HPV infections, these cells exhibit an impaired phenotype (281–286). For instance, lower numbers and frequencies of LCs (281–283), changes in their morphological characteristics (284), and decreased capacities to induce a proper immune response have been reported in this context (286, 287). In light of all this, the critical role that LCs play during infections, and the persistence of HPV infection is more than evident. Therefore, further studies and insights in the role of these cells during this disease are mandatory to elucidate and suggest new approaches for its treatment.

### Adenovirus

Adenovirus (AdV) are non-enveloped viruses responsible for diseases associated with the upper and lower respiratory tract, reaching even the gastrointestinal tract and the conjunctiva and cornea, depending on the species of the AdV and the immune condition of the host (288). Considering all this, it is important to describe how the innate immune response, and particularly DCs, are capable of detecting and responding once AdV reaches an organism, although the mechanisms associated with this are not completely well understood.

AdV targets of infection are both dividing and quiescent cells, with specific tropism associated with the species of the virus (289). In this line, DCs can be transformed with AdV, with entry mechanisms that differ from those of epithelial cells, and with the capability of being modulated by cytokines and chemokines (288–290). Remarkably, it has been described that AdV is capable of inducing maturation of DCs strongly upon encounter (290, 291). Moreover, studies indicate that AdV presented as immunocomplexes to DCs can induce pyroptosis -a type of apoptosis associated with the formation of the inflammasome- in these cells (291).

It has been described that AdV can be recognized by TLR2, TLR4, and TLR9 (292–295). Engagement of TLR2 during AdV has been shown to be necessary for the activation of NF-κB and the mount of an effective humoral response, as KO mice for these receptors show deficiencies in these responses (294). Accordingly, TLR4 activation -by AdV complexes- leads to the IL-1β-associated inflammatory response, possibly modulating the formation of the inflammasome that will induce pyroptosis (295). Finally, the recognition of TLR9 in PBMCs and pDCs leads to the secretion of many pro-inflammatory cytokines and the activation of the type I IFN pathway (292, 293). Since all these receptors require the adaptor protein MYD88 to properly signalize, the upregulation of this protein has been described upon infection (296, 297). Besides these TLRs, AdV can also be recognized by other PRRs, such as Lectin receptors -for instance, Siglecs and Galectins (298, 299)- and inflammasome-associated DNA sensings receptors -such as NLRs AIM2 and NALP3 for AdV (300, 301). Activation of all these receptors will induce maturation and activation of DCs, leading to the mounting of a classical antiviral response. However, further studies are required to properly elicit the role of DCs in the infection of this virus.

### Hepatitis B Virus

Hepatitis B virus (HBV) is the etiological agent responsible for either a self-limiting or a chronic infection that affects the hepatocytes in the liver of over 250 million people worldwide (302, 303). The disease caused by this virus is characterized by an inadequate immune response and, in the long-term, the development of hepatitis, cirrhosis, and hepatocellular carcinoma (302). The interaction between this virus and the immune system is both innate- and adaptive-related, although the role of the latter has been more thoroughly described than the former (194, 207, 302). Accordingly, the adaptive immune response seems to play a more significant role in the modulation of chronic infection (194, 207, 302). In this point, DCs are fundamental since they work as the bridge between the innate and the adaptive response.

HBV induces an inefficient innate immune response -and also an impaired activation of DCs-, due to several of its characteristics and its infectious cycle (207). For instance, most of its DNA is recognized just as host genetic material -therefore not triggering an immune response-, although certain conformations of the HBV's DNA may be recognized by RIG-I or TLR9, as described by some authors (205–207). Accordingly, acute exposure of this virus does not induce the secretion of IFN or pro-inflammatory cytokines by either pDCs (the responsible for secreting type I IFN) or other immune cells, therefore not mounting the first line of defense against viral infection (207, 304). Even so, increased secretion of IL-10 upon infection has been reported, which could explain the lack of response described for this virus, as this cytokine will induce a state of tolerance in the surveilling immune cells (305, 306). Remarkably, since it has been described that pDCs from HBV infected patients are impaired in their capacities to secrete IFN, this also results in a decreased capacity to induce the proper activation of NK cells (302, 307).

Recognition of PAMPs derived from HBV by PRRs -and therefore the innate immune system and DCs- is currently a controversial field, as *in vitro* studies of HBV are hard to perform. These difficulties are associated with the high multiplicity of infection (MOI) required to achieve a productive infection in human primary hepatocytes (308). Moreover, several other factors difficult *in vitro* studies with this virus, such as an inadequate capacity of this virus to diffuse in monolayers (308), the differences in the inoculum used, and the generation of artifactual results obtained from all these points (207, 308). Despite all this, it has been suggested that the nucleocapsid of this virus may act as a TLR2 ligand, leading to the secretion of several pro-inflammatory cytokines (309, 310). Also, Vanlandschoot et al. reported that subviral particles might be recognized by one of the co-receptors of TLR4, inducing the activation of myeloid cells (311, 312). Therefore, more studies are required to elicit further the role of DCs and the innate response during an HBV infection.

A study performed recently by Yonejima et al. analyzed the gene expression and function of DCs in a cohort of 64 humans infected with HBV (313). In this article, the authors show that there are no differences in the number of circulating DCs in the PBMCs samples of the subjects, as previously reported for HBV infected patients (314, 315). Accordingly, no differences in the levels of expression of CD80, CD83, CD40, and CCR7 were found in the cDCs subset analyzed. However, DCs obtained from these patients exhibited impaired antigen-presenting capacities, a decrease in their capacities to migrate, and impaired levels of cytokine production, as seen during *in vitro* assays. Other studies have also shown similar results, still supporting the notion that HBV induces a tolerogenic state (316, 317). By performing microarray assays, the authors also suggest that one of the genes responsible for this impairment is the IL-6 signal transducer (IL6ST), since rescuing the proper expression levels of this gene resulted in the recovery of the previously indicated function of DCs. Thus, IL6ST may constitute a possible therapeutic target to treat HBV infection (313).

Indeed, DCs seems to play a pivotal role during the self-limiting and the chronic disease caused by HBV, but further researches are required in order to thoroughly comprehend the magnitude of these cells in this disease.

### Herpesvirus

The order *Herpesvirales* comprises many species of herpesviruses, among which we can highlight the human alphaherpesvirus 1 and 2 -commonly known as human herpes simplex virus 1 and 2 (HSV-1 and HSV-2)-, the human alphaherpesvirus 3 -commonly known as varicella-zoster virus (VZV)- and the human gammaherpesvirus 4 -commonly known as Epstein-Barr virus (EBV) (318). All these viruses, among others of the same order, are responsible for different diseases in humans, with symptoms ranging from blisters to severe neurological alterations (319).

As stated above, human alphaherpesvirus comprises HSV-1 and HSV-2. Upon HSV infection, and as expected for most viruses, the type I IFN pathway is activated, leading to the secretion of IFN-α and IFN-β (320, 321). This activation is mediated by PRRs that can recognize HSV, such as TLR3 and TLR9, which will be activated in the presence of viral-related genetic material. Moreover, TLR2 and TLR4 have also been described to be activated upon HSV infection (322). RIG-I will detect viral-related genetic material -particularly dsRNA- and induce the production of type I IFN and other proinflammatory cytokines (321). Remarkably, it has been described that US11, an HSV-1 tegument protein, is capable of inhibiting the antiviral protein elicited upon RIG-I activation by degrading downstream signals of this pathway (323). Since most of these PRRs are expressed in DCs, they are relevant in their role for the modulation of this infection. Remarkably, HSV-1 is capable of infecting DCs, and HSV-infected DCs are not capable of achieving maturation (324). Despite this, HSV-infected and non-matured DCs are capable of secreting proinflammatory cytokines, which will help other DCs to achieve maturation if they are not infected (324). Once matured, DCs will be able to internalize the virus, either as a free particle or by phagocyting other infected cells -even infected DCs-, degrade it, and eventually cross-present it to the adaptive immune system, which will initiate a new response (321, 324). Furthermore, depletion of the IgD glycoprotein in HVS-2 has been found to promote efficient activation of DCs and effective activation of CD4^+^ and CD8^+^ T cells (325).

The main target of infection and replication of human alphaherpesvirus 3 or VZV are epithelial cells of the respiratory tract. Initial viral replication will eventually lead to viremia and the characteristic rash -commonly known as chickenpox- that is particular for the disease caused by this virus (326). Upon resolution of this stage, the virus achieves latency in the nerve cells that innervate the portions of skin affected with the rash (326). In order to be reactivated, the virus must be mobilized by anterograde transport into the skin cells. As seen for HSV, DCs in the skin can be infected by VZV -as reported in *ex vivo* experiments (327). Moreover, infection by VZV induces a change in the repertoire of DCs available in the zone of the skin affected, which could be mainly related to the movement of these cells from and into the LN (327). It may be significant to highlight that the repertoire of DCs commonly detected in the skin are cDCs and LCs, and, after infection, monocyte-derived inflammatory DCs and pDCs are found (326–328). Accordingly, and as seen for HSV, DCs are capable of mounting a primary antiviral response upon VZV infection, therefore prompting the secretion of type I IFN and other proinflammatory molecules. However, VZV infection of DCs can be one of the mechanisms that this virus could use to disseminate in the organism (326).

Finally, human gammaherpesvirus 4 or EBV has been reported to infect over 90% of the world population, achieving latency and persistence in its host (329). This virus is associated with many diseases in humans, among which the appearance of tumors can be included -Hodgkin's lymphoma is one of the most remarkable among these (330). Surprisingly, EBV can also infect DCs, as seen for HSV and VZV (330). This virus can also be recognized by TLR3 and TLR9, therefore leading to the activation of the type I IFN pathway (331). The activation of this pathway has been shown to render B cells less prone to transformation induced by this virus, therefore partially restricting the symptoms of this disease (332). This environment will also induce the activation of other innate cells, such as NK cells and PMNs, that will aid in the control and clearance of this virus (331).

## Conclusions

In this article, we have stated the significant part that DCs play during important infectious diseases that affect humans. Not only are they effector phagocytic cells that can contribute to IFN-mediated antiviral responses, but they must also act as a bridge between the innate and adaptive immune response, activating lymphocytes, the key players of the adaptive response, so that these cells can effectively face the respective viral infection. DCs must perform this by recognizing either PAMPs or DAMPs through their vast arrays of PRRs, which will guide DCs -and another cells type- to the correct effector profile. Triggering of PRRs will induce signaling cascades that will eventually promote the secretion of several molecules with immune-related roles, such as cytokines and chemokines. Interferon is one of the most important cytokines related to the antiviral response, key to efficient clearance of the viruses from the hosts. Although several types of cells are capable of activating the type I IFN pathway -and therefore secreting this molecule-, DCs have a fundamental role in this process. They are strongly associated with the secretion of this cytokine, as they can recognize different components of the virus to activate this antiviral response efficiently, through the previously mentioned PRRs. Therefore, further therapeutic approaches against the discussed pathogens will require to focus on the role of these cells in order to effectively promote an immune response that comprises both the innate and adaptive branch of the immune system.

## Author Contributions

JS: conceptualization, writing original draft, review, editing, and revision. NG: writing original draft, review, editing, and revision. KB: writing original draft. CA: figure design, review, and revision. GP: writing original draft, review, and revision. RB: writing original draft. SB: editing and revision. AK: conceptualization, revision of original draft, editing, and revision. All authors listed have made substantial, direct, and intellectual contribution to the work and approved it for publication.

## Conflict of Interest

The authors declare that the research was conducted in the absence of any commercial or financial relationships that could be construed as a potential conflict of interest.
